# Are FoMO, Experiential Avoidance, and Emotional Distress Related to Problematic Social Network Use in Young Adults?

**DOI:** 10.3390/healthcare13222988

**Published:** 2025-11-20

**Authors:** Isabel C. Salazar, Raquel Santamaría-Perales, Ana M. Cuevas-Toro

**Affiliations:** Department of Personality, Evaluation and Psychological Treatment, Faculty of Psychology, University of Granada, University Campus Cartuja, 18011 Granada, Spain; isabelsalazar@ugr.es (I.C.S.); raquelstmp@correo.ugr.es (R.S.-P.)

**Keywords:** problematic social network use, fear of missing out, experiential avoidance, anxiety, depression, stress

## Abstract

**Background/Objectives**: Social networks have brought exciting possibilities for interacting with others in real time, anywhere in the world. However, problematic social network use (PSNU) causes distress and dysfunction in daily life. Young people may be vulnerable due to their high degree of digital connectivity and the particularities of psychosocial development. The primary aim of this study was to assess the presence of PSNU and its relationship with anxiety, depression, stress, fear of missing out (FoMO), and experiential avoidance in young people, while also examining gender differences. **Methods:** An online survey was conducted with a non-probabilistic sample of 219 young people between the ages of 18 and 25 (*M* = 20.50, *SD* = 2.42; 74.4% women), which included self-report measures of the aforementioned variables. **Results:** A total of 27.4% reported PSNU, but there were no differences by gender. PSNU was positively and significantly related to all the variables analyzed, with the highest correlations being with FoMO and experiential avoidance, especially in women. Regression analysis showed that the set of variables explains 17.2% of the variance in PSNU, but only FoMO contributed positively and significantly to PSNU in the overall sample and in women, but not in men. **Conclusions:** This is the first study to jointly compare the predictive power of key variables (anxiety, depression, stress, experiential avoidance, and FoMO) on PSNU in young adults. Additionally, we examined gender differences and utilized validated instruments. Our results show that only FoMO plays a relevant role in accounting for PSNU variance, although more so in women than in men. Also, the scores in experiential avoidance are significantly higher in women compared with men. These results support the idea that PSNU may serve as a strategy for avoiding distress, specifically FoMO, particularly in women. In terms of clinical implications, it would be highly interesting to analyze the ways and contexts in which social media could be used in a healthier manner and in alignment with personal values.

## 1. Introduction

The expansion of (and access to) the internet, the development of smartphones, and social network platforms such as Instagram, X, TikTok, and Facebook, have made social network use a common behavior. In 2024, there were 5.4 billion social network users, with northern and western Europe having the highest social network penetration rates, followed by southern Europe in third place [1]. Social networks have become a source of information (and misinformation also), communication, social and work relationships, and entertainment/leisure [2]. Nevertheless, excessive social networks use has been associated with negative physical and psychological effects [3,4]. This occurs because people spend a lot of time on this activity and neglect or delay other activities that are important in their daily lives (e.g., academic, work or household responsibilities, social relationships, other forms of leisure time, etc.) and they experience emotional distress (e.g., anxiety, low mood, irritability) when they reduce their social network use or are unable to access them. In these cases, we refer to a problematic social network use (PSNU) [4] or social networks addiction (SNA). A prevalence of 24% for SNA has been reported in 32 countries and 7 different regions [5], suggesting that this is a growing global public health problem.

Among young adults (aged 18 to 25), social network use is widespread [1] and has become a key factor in the development of their identity, as well as their social and emotional development [6]. It has been observed that young adults appear to be increasingly affected by PSNU, and that this problem is significantly more prevalent compared to those over the age of 25 [4,7] and 23% of those with PSNU are university students [5].

Regarding gender differences in PSNU/SNA, we find that the situation is controversial. Some studies indicate that women may be the most vulnerable group [4,8]. For example, it has been reported that women score higher than men on SNA (*F* = −1.71, *p* < 0.05) and on excessive social networks use (*F* = −2.33, *p* < 0.01). However, regression analyses did not indicate that gender acts as a predictor variable for SNA, but it did for excessive social networks use (explaining 1.5% of the variance; *R*^2^ = 1.5%, β = 0.122, *p* = 0.02) [4]. This same study noted that women had significantly higher scores than men on the two aspects that, according to the authors, form part of the PSNU: negative social comparison (*p* = 0.003) and addictive consequences (*p* = 0.016). In contrast, we have found some studies [9,10,11] and meta-analyses [5,12] that indicate that there are no gender differences with respect to PSNU/SNA.

Some psychological aspects have been associated with PSNU/SNA. These include depression, anxiety, and stress, as well as the fear of missing out (FoMO) [12]. FoMO is perhaps the most studied variable and is understood as the constant concern about being disconnected from social experiences that others may be enjoying online. FoMO is linked to SNA and has been identified as the strongest predictor of SNA among adolescents [13], because people who experience FoMO have an increased tendency to utilize social networking platforms [14,15]. In university samples, evidence indicates that FoMO is related to SNA (0.37 ≤ *r* ≤ 0.73) [10,16,17,18], FoMO can also predict PSNU (β = 0.457, *p* < 0.001) [16] and SNA (β = 0.61, *p* < 0.001) [18], or act as a mediator between sensation seeking and SNA [17] as well as between stress perception and PSNU [10]. Relationship between FoMO and SNA occurred significantly in men (*r* = 0.77, *p* < 0.01) and women (*r* = 0.68, *p* < 0.01), but this relationship was statistically greater in men than in women (*z* = 2.354, *p* = 0.009) [18]. Regarding studies of the general population, a relationship has been reported between FoMO and social media use (*r* = 0.33, *p* < 0.01) [19] and between FoMO and SNA (β = 0.46, *p* < 0.001) [20], as well as between FoMO and PSNU (*r* = 0.50, 95% CI [0.46, 0.53]) [21].

Experiential avoidance is another psychological aspect investigated in relation to PSNU/SNA. Experiential avoidance is understood as the tendency to reject, escape, or attempt to control unpleasant internal experiences (e.g., thoughts, emotions, memories) through various behaviors [22]. Regarding compulsive social media use, experiential avoidance can represent a dysfunctional emotional coping strategy, used to mitigate feelings of emptiness or personal dissatisfaction [23]. Nevertheless, evidence on this relationship between experiential avoidance and PSNU/SNA is scarce and contradictory. A study of a general population sample reports that experiential avoidance is associated with the abusive social networks use (*r* = 0.38, *p* < 0.01) and that in women this relationship is more unfavorable (*r* = 0.45, *p* < 0.01) than for men (*r* = 0.17, *p >* 0.05) [24]. While another study reports that there is no direct relationship between social network use and experiential avoidance, but this relationship appears to be conditioned by mobile phone addiction [25].

With regard to the relationship between anxiety and the PSNU/SNA, studies indicate that in adults in the general population, the relationship is significant (0.22 ≤ *r* ≤ 0.46) [3,8,24], in both women (*r* = 0.37, *p* < 0.01) and men (*r* = 0.30, *p* < 0.01) [24]. The relationship between the two variables is similar in a university sample (*r* = 0.23, *p* < 0.01) [4].

Studies on the relationship between depressive symptoms and PSNU/SNA in the general adult population indicate a significant correlation between the two variables (0.21 ≤ *r* ≤ 0.46) [3,8]. Likewise, it has been reported that those with more depressive symptoms have a higher risk of developing SNA (*F* = 13.33, *p* < 0.01) [7].

Regarding the relationship between stress and PSNU/SNA, studies (mostly with university samples) indicate that the perception of stress is significantly related to PSNU/SNA (0.29 ≤ *r* ≤ 0.38) [9,10,16], adding that a higher level of stress predicts more severe SNA (β = 0.17, *p* = 0.001) [9]. In one study, path analyses revealed that there was no direct effect of stress on PSNU (β = 0.050, *p >* 0.05), but there was an indirect effect through FoMO (effect = 0.18, 95% CI: [0.12, 0.23]) [16]. Contrary to these findings, another study states that there was no relationship between perceived stress and the negative impact of WhatsApp use among university students [26].

Given this state of the art, we can highlight how previous research has analyzed variables such as FoMO, experiential avoidance, and psychopathological aspects (anxiety, depression, and stress) in relation to PSNU/SNA separately and many of the studies are based on samples of adults. FoMO has the strongest evidence of its association with and predictive power in PSNU, experiential avoidance has been the least studied in terms of its relationship with PSNU, and with regard to the other symptomatic variables, there are no conclusive results, as there is evidence both for and against their association with PSNU. We have not found any studies that jointly compare the predictive power of these variables for PSNU in young adults.

Therefore, the goals of this study are as follows: (1) To explore the possible gender differences in terms of PSNU, anxiety, depression, stress, FoMO, and experiential avoidance in a group of young adults; (2) to analyze the degree of relationship between PSNU and the other psychological variables globally and by gender; (3) to identify which of these variables predict PSNU in these young adults and determine whether these differ by gender; and (4) to analyze the differences in hours per day of social networks use, anxiety, depression, stress, FoMO, and experiential avoidance, comparing young adults with and without PSNU.

We propose the following hypotheses: (1) there are gender differences in the various psychological aspects evaluated, with women being the most affected; (2) PSNU is positively and significantly related to the other psychological variables; (3) the psychological variables evaluated will contribute jointly to PSNU, although there will be greater variance explained in women than in men; and (4) there are significant differences between the group with PSNU and the group without PSNU in the variables evaluated.

## 2. Materials and Methods

### 2.1. Study Design

This is a cross-sectional, descriptive, and correlational study [27].

### 2.2. Study Sample

A total of 219 Spanish nationals between the ages of 18 and 25 (*M* = 20.50, *SD* = 2.42) participated in the study. Of the total sample, 163 were women (*M* = 20.28, *SD* = 2.19) and 56 were men (*M* = 21.15, *SD* = 2.27), with significant differences in age according to gender. In terms of educational level, 60.2% had a university degree (undergraduate, master’s, or doctorate), with no differences observed according to gender. The average number of hours per day of social network use (Monday to Friday) was 4.07 for the total sample, 4.10 h for men and 4.06 h for women. The average number of hours per day of social network use during the weekend was 4.87 for the total sample, 5.06 h for men and 4.91 h for women (Table 1).

### 2.3. Instruments

An online survey was designed with all the self-report measures indicated below, preceded by a section to collect sociodemographic data (age, gender, educational level, nationality, and number of hours per day spent using social media (from Monday to Friday and during the weekend, separately).

The Social Networks Addiction Test (“Test de Adicción a las Redes Sociales”, TARS) [7]. TARS assesses problematic use and addiction to social networks. It consists of 36 items with a dichotomous response format (1 = “True”/0 = “False”). The total score is obtained by adding the direct responses to the items, except for item 24, for which the score is reversed. The scores were classified based on the decatypic scale reported by the authors. A score ≥ 13 (decatype 8) indicates the presence of PSNU [7]. TARS has an unifactorial structure, with adequate fit indices (χ^2^/*df* = 2.74; goodness-of-fit index [GFI] = 0.94) and high internal consistency (Cronbach’s α = 0.90) [7]. In our study, Cronbach’s alpha was 0.85 and McDonald’s Omega was 0.85.

Spanish version [14] of the Fear of Missing Out (FoMO) [28]. This self-report measure allows us to identify the degree to which a person fears being left out of rewarding experiences that others may be having and that are happening on social networks. It consists of 10 items with a 5-point Likert-type response format (from 1 = “Not at all” to 5 = “Very much”). The total score is obtained by adding direct responses to the items. The higher the score, the greater the FoMO. The structure of the instrument is unifactorial and has shown good internal consistency (Cronbach’s α = 0.85). In our study, Cronbach’s alpha was 0.89 and McDonald’s Omega was 0.89. This instrument demonstrated measurement invariance between men and women [29].

Spanish version [30] of the Acceptance and Action Questionnaire-II (AAQ-II) [31]. AAQ-II assesses the difficulty some people have in accepting negative thoughts and emotions, as well as their tendency to avoid these experiences even when they interfere with their life goals. It consists of 7 items, with response options on a 7-point Likert scale (from 1 = “Never true” to 7 = “Always true”). The total score is obtained by adding direct responses to the items; the higher the score, the greater the level of experiential avoidance. The AAQ-II is a unifactorial self-report measure with adequate internal consistency (Cronbach’s α = 0.88). In our study, Cronbach’s alpha was 0.93 and McDonald’s Omega was 0.93. This instrument demonstrated measurement invariance between men and women [32,33].

Spanish version [34] of the Depression, Anxiety, and Stress Scales (DASS-21) [35]. DASS-21 assesses the symptoms associated with anxiety, depression, and stress separately. It consists of 21 items, organized into three subscales of 7 items each. Responses are recorded on a 4-point Likert scale (from 0 = “Nothing has happened to me” to 3 = “It has happened to me a lot or almost always”). A score is obtained for each subscale by adding up the direct responses to the items in each one. The higher the score, the greater the anxiety, depression and stress, respectively. The subscales have acceptable levels of reliability, with Cronbach’s alpha coefficients between 0.73 and 0.93 and 0.90, respectively [34,36]. In our study, Cronbach’s alphas were 0.80, 0.90, and 0.88 for the Anxiety, Depression, and Stress subscales, respectively, and McDonald’s Omega were 0.80, 0.90 and 0.87 for the anxiety, depression, and stress subscales, respectively. This instrument demonstrated measurement invariance between men and women [37].

### 2.4. Procedure

The online survey was designed on the LimeSurvey platform, using the last author’s official university email account, ensuring that only the authors of the study had access to the data. The first page included information about the study’s goals and the conditions for participating in it. Participants were then asked to confirm that they had read and agreed to participate voluntarily in the study. This was followed by the sociodemographic data sheet and then the instruments used to measure the variables of interest. The survey ended with a thank you for participating and a contact email address for any questions about research or data processing.

The data were collected in April 2025 using non-probabilistic sampling with the snowballing technique, employing social networks (Instagram, WhatsApp, and X) and the university’s Moodle platform. Subsequently, the database was organized in Excel and reviewed and cleaned. Cases in which the age exceeded 25 years or any of the questionnaires had more than 20% of their items unanswered were eliminated. Additionally, the scores for item 24 of the TARS were reversed.

This study was approved by the Human Research Ethics Committee of the University of Granada in March 2025 under number 4820/CEIH/2025.

### 2.5. Data Analysis

Data analysis was performed using SPSS v. 30. Descriptive statistics of the variables were used, and possible gender differences were evaluated using Student’s *t*-test and chi-square, depending on the type of variable. Previously, we verified the assumptions of normality and homoscedasticity using the Kolmogorov–Smirnov test and Levene’s test, respectively. The degree of relationship between the variables was calculated using Pearson’s *r* (*p* ≤ 0.05), both for the total sample and by gender. Multiple regression analyses were then performed, both with the overall sample and by gender, to establish the joint contribution of the predictor variables for PSNU, at a 95% confidence level. Finally, two groups were defined, PSNU vs. WPSNU, based on score ≥ 13 (decatype 8) and score ≤ 12 (decatype 7) in the TARS, respectively, to describe both groups. Possible differences between groups were examined using Student’s *t*-test and the effect size (Cohen’s *d*). Previously, we verified the assumptions of normality and homoscedasticity using the Kolmogorov–Smirnov test and Levene’s test, respectively.

## 3. Results

### 3.1. PSNU, FoMO, Experiential Avoidance, Anxiety, Depression, and Stress

Prior to conducting the statistical analyses, the normality assumption for all analyzed variables was tested using the Kolmogorov–Smirnov test. The results indicated that all variables, except for experiential avoidance (*Z* = 0.059, *p* = 0.067), violated the normality assumption: PSNU (*Z* = 0.209, *p* ≤ 0.001), FoMO (*Z* = 0.105, *p* ≤ 0.001), anxiety (*Z* = 0.175, *p* ≤ 0.001), depression (*Z* = 0.188, *p* ≤ 0.001) and stress (*Z* = 0.087, *p* ≤ 0.001). Nonetheless, given the large sample size (*N* = 219), the use of parametric tests is still permissible. Regarding Levene’s test, the *p*-value for all variables was greater than 0.05, thus confirming the assumption of homogeneity of variances was met. Consequently, parametric tests will be used for the statistical analyses concerning this objective.

Table 2 presents mean and standard deviation of the total sample and by gender in PSNU, FoMO, experiential avoidance, anxiety, depression, and stress. In addition, the results of Student’s *t*-test used to explore gender differences are shown. Significant differences were found between men and women in experiential avoidance and stress, with higher scores in the women’s group, and a small effect size (Cohen’s *d*).

### 3.2. Relationships Between PSNU and the Other Psychological Variables Evaluated

Next, we calculated Pearson’s correlations between PSNU, FoMO, experiential avoidance, anxiety, depression, and stress. In the total sample, we found PSNU correlates positively and significantly with all the variables analyzed, with the exception of stress. The highest correlations were with FoMO (*r* = 0.428) and experiential avoidance (*r* = 0.217) (Table 3).

The correlational analysis for each gender shows that in the case of women, PSNU correlates positively and significantly only with FoMO (*r* = 0.441) and experiential avoidance (*r* = 0.164) (Table 4).

In the case of men, PSNU correlates positively and significantly with all variables except stress, with the highest correlations being with FoMO (*r* = 0.392) and experiential avoidance (*r* = 0.369) (Table 5).

### 3.3. Regression Analysis for Problematic Social Networks Use

To identify the contribution of the psychological variables evaluated in predicting PSNU in this sample of young adults, we performed multiple regression analyses using the Enter method to reduce type I error.

For the regression analysis with the total sample (*N* = 219), we included all predictor variables that correlated positively and significantly with PSNU (Table 3). The relationship between PSNU and FoMO, experiential avoidance, anxiety and depression is moderate (multiple *R* = 0.432). Together, FoMO, experiential avoidance, anxiety and depression explain 17.2% of the variance in PSNU (*R*^2^_adjusted_ = 0.172). ANOVA showed that we could predict PSNU with the variables introduced, *F*_(4,214)_ = 12.293, *p* = 0.001. Also, the effect size is medium (f^2^ = 0.207). Table 6 shows the regression coefficients for the predictor variables. The standardized regression coefficients (β) indicate that FoMO has the greatest predictive power of PSNU (β = 0.404). This effect is substantially stronger than that of the other variables in the model as an experiential avoidance (β = 0.053), depression (β = 0.028) and anxiety (β = −0.017). Finally, model diagnostics confirm the robustness of the analysis. To check the assumptions of homoscedasticity and model linearity, we include the Scatter Plot (Figure S1) in the Supplementary Materials. To check the assumption of residual normality, we include the Normal Q-Q Plot (Figure S2) in the Supplementary Materials.

For the regression analysis with women (*n* = 163), we included FoMO and experiential avoidance, as these were the two variables that showed a significant correlation with PSNU (Table 4). The relationship between PSNU and FoMO and experiential avoidance is moderate (multiple *R* = 0.442). Together, FoMO and experiential avoidance explain 18.5% of the variance in PSNU (*R*^2^_adjusted_ = 0.185). ANOVA showed that we could predict PSNU with the variables introduced, *F*_(2,160)_ = 19.390, *p* = < 0.001. Also, the effect size is medium (f^2^ = 0.227). Table 7 shows the regression coefficients for the predictor variables. The standardized regression coefficients indicate that only FoMO contributes positively and significantly to PSNU. FoMO has the greatest predictive power of PSNU (β = 0.447), while experiential avoidance (β = −0.015) shows an absence of independent statistical significance. Finally, model diagnostics confirm the robustness of the analysis. To check the assumptions of homoscedasticity and model linearity, we include the Scatter Plot (Figure S3) as Supplementary Materials. To check the assumption of residual normality, we include the Normal Q-Q Plot (Figure S4) as Supplementary Materials.

For the regression analysis with men (*n* = 56), we included all predictor variables except Stress, which had no significant correlation with PSNU (Table 5). The relationship between PSNU and FoMO, experiential avoidance, anxiety, and depression is moderate (multiple *R* = 0.474). Together, FoMO, experiential avoidance, anxiety, and depression explain 16.4% of the variance in PSNU (*R*^2^_adjusted_ = 0.164). ANOVA showed that we could predict PSNU with the variables introduced, *F*_(4,51)_ = 3.69, *p* = 0.010. Also, the effect size is medium (f^2^ = 0.196). Table 8 shows the regression coefficients for the predictor variables. The standardized regression coefficients indicate that no variable contributes significantly to PSNU, although FoMO exhibits the greatest relative contribution (β = 0.277). Finally, model diagnostics confirm the robustness of the analysis. To check the assumptions of homoscedasticity and model linearity, we include the Scatter Plot (Figure S5) as Supplementary Materials. To check the assumption of residual normality, we include the Normal Q-Q Plot (Figure S6) as Supplementary Materials.

### 3.4. Differences Between the PSNU Group and the WPSNU Group

Before making comparisons between the group with PSNU (decatype ≥ 8; *M*_TARS_ = 17.87, *SD* = 4.31) and WPSNU (decatype ≤ 7; *M*_TARS_ = 9.51, *SD* = 3.24), we ensured that they were equivalent and that there were no differences in terms of the socio-demographic variables collected (age, gender, and level of education) (*p >* 0.05).

Prior to conducting the statistical analyses, the normality assumption for all analyzed variables was tested using the Kolmogorov–Smirnov test. The results indicated that all variables, except for experiential avoidance (*Z* = 0.061, *p* = 0.065), violated the normality assumption; hours per day of social network use (Monday to Friday) (*Z* = 0.140, *p* ≤ 0.001), hours per day of social network use (during the weekend) (*Z* = 0.138, *p* ≤ 0.001), FoMO (*Z* = 0.106, *p* ≤ 0.001), anxiety (*Z* = 0.187, *p* ≤ 0.001), depression (*Z* = 0.209, *p* ≤ 0.001) and stress (*Z* = 0.091, *p* ≤ 0.001). Nonetheless, given the large sample size (*N* = 219), the use of parametric tests is still permissible. Regarding Levene’s test, the *p* value for all variables was greater than 0.05, thus confirming the assumption of homogeneity of variances was met. Consequently, parametric tests will be used for the statistical analyses concerning this objective.

Differences in hours per day of social network use (Monday to Friday), in hours per day of social network use (during the weekend), FoMO, experiential avoidance, anxiety, depression, and stress between the PSNU and WPSNU groups were established using Student’s *t*-test (*p ≤* 0.05). Significant differences were found between the two groups in hours per day of SNU (Monday to Friday and during the weekend), FoMO, and experiential avoidance, with higher scores in the PSNU group compared to the WPSNU group (Table 9). Effect sizes in these variables were moderate with the exception of experiential avoidance, which had a small effect size.

## 4. Discussion

PSNU is becoming a major public health problem [5]. This study had several goals. The first goal was to explore the possible gender differences in PSNU, anxiety, depression, stress, FoMO, and experiential avoidance in a group of young adults. In this regard, we found that hypothesis 1 is partially fulfilled. The results indicated that women have significantly higher scores than men in experiential avoidance and stress; however, there are no differences in the other variables (PSNU, FoMO, anxiety, and depression). These data are consistent with some results of previous studies [9,10,18,19,24], but not with others [4,10,18,24,38]. This disparity in results may be due to the different instruments used, the different proportions of women and men in the samples (with a higher proportion of women in almost all studies), cultural differences and the average age of the participants (most studies evaluate adults).

The second goal of this study was to analyze the degree of relationship between PSNU and other psychological variables globally and by gender. In this regard, we found that hypothesis 2 is indeed fulfilled, especially in the total sample and in men. In the women sample, PSNU correlates positively and significantly only with FoMO and experiential avoidance. What is interesting about these results is that, although, in general, there are positive and significant correlations between PSNU and the other variables (anxiety and depression), FoMO and experiential avoidance are the variables with the highest positive correlation in the total sample, and in both women (where the other variables do not correlate) and men. FoMO is a type of anxiety related to PSNU and occurs in different types of samples, adolescents [12], young adults [10,16,17,18], and the general population [19,20]. Regarding experiential avoidance, our results coincide with a study (which also used the TARS) that found a relationship between experiential avoidance and PSNU, especially in women [24]. Likewise, we found this relationship between experiential avoidance and mobile phone abuse [25]. This suggests that FoMO and experiential avoidance are the variables that show the strongest relationship with PSNU in young adults.

The third goal of this study was to identify which of these variables account for the variance in PSNU in these young adults and determine whether they differ according to gender. In this regard, we found that hypothesis 3 is supported. In the women sample, FoMO and experiential avoidance together explain 18.5% of the variance in PSNU, although only FoMO contributes positively and significantly to PSNU. In the men sample, FoMO, experiential avoidance, anxiety, and depression together explain 16.4% of the variance in PSNU, but no variable contributes significantly to PSNU. From these results, we highlight that only FoMO contributes positively and significantly to explaining the variance in PSNU in the total and in women samples, but not in men sample, as is reported in other studies with adolescents [13]. We have not found any studies that jointly compare the predictive power of such relevant variables as anxiety, depression, stress, experiential avoidance, and FoMO on PSNU in young adults, hence the relevance of our study. In a similar study, the results differed from ours in terms of the variables that account for the variance in PSNU according to gender [24]. It was noted that experiential avoidance (AAQ-II) and low sense of meaning explain the variance in PSNU in women and that anxiety explains the variance in PSNU in men. This disparity in results can be explained by the difference in the mean age of the samples and by the instruments used to assess anxiety.

In terms of gender differences, our results suggest that FoMO and experiential avoidance are relevant variables for understanding PSNU in young adult women. A plausible explanation for it may have to do with negative social comparison, which is more prevalent in women than in men [8] and has been linked to FoMO [20]. Cultural and social pressures regarding physical appearance and social status disproportionately affect women more than men [21], leading to greater negative social comparison in women [4]. Also, women tend to engage in social media use in a more active and emotional manner, which could contribute to an increase in PSNU [21]. Specifically, women tend to seek emotional support and self-expression on social media to a greater extent than men [21]. While this may serve as a momentary relief from emotional distress, relying on social media use as the sole coping mechanism for discomfort over the long term can evolve into yet another experiential avoidance strategy [24].

The fourth goal was to analyze the differences in hours per day of social media use (Monday to Friday and during the weekend), anxiety, depression, stress, FoMO, and experiential avoidance between young adults with PSNU and those without this problem (WPSNU). In this regard, we found that hypothesis 4 is partially fulfilled. Our data indicate that the differences are significant in hours per day of SNU (Monday to Friday and during the weekend), FoMO, and experiential avoidance, with higher scores in the PSNU group than in the WPSNU group. Taking into account the differences in this variable, it would be interesting to investigate other factors that would allow PSNU to be characterized, such as nighttime use, passive use, reasons for use, number of social media use, or addiction to ‘likes’ [12]. The relevance of the number of hours people spend on social media use has to do with the fact that use becomes problematic when it interferes with a person’s daily activities [4] or when the person uses them as a fundamental mechanism to relieve stress, loneliness, or depression [18]. Furthermore, our study indicates that the transition from healthy social networks use to PSNU in young adults may be related to high levels of FoMO and high levels of experiential avoidance. Thus, PSNU can be understood as a strategy for avoiding discomfort [24,25]. This emotional discomfort does not correspond to the usual symptoms of stress, anxiety, or depression, but rather to a very specific anxiety, FoMO [10,17,19,28,39]. This type of anxiety is relatively new, as it did not exist before social media and has been fostered by them [21]. Apparently, FoMO is not only the fear of missing out on other people’s events, but also the anxiety of missing out on information about the dynamics and trends of social media [21]. Thus, people with a greater fear of missing out on these experiences will tend to connect more to social media [14,15].

As for the limitations of our study, first, as it is a cross-sectional and correlational study, we cannot point to causal relationships between psychological variables and PSNU, making this a possible line of future research [12]. Second, our sample has a much higher proportion (74%) of women than men. Although our a priori G*Power (Version 3.1.9.7) analysis aimed for *n* = 88 in the male group, this was not achievable, so comparative analyses should be taken with caution. Third, this is a non-probability sample, so the results cannot be generalized to the general population. Fourth, exclusively using self-reports instruments might heighten the risk of social desirability bias. The limitations discussed (namely, the gender imbalance in our sample and its non-probabilistic nature) might affect the statistical power and the generalizability (or external validity) of the results. Future research should strive to obtain a larger and more evenly distributed sample by gender.

In terms of clinical implications, we suggest that total restriction of social media is not necessarily the only solution to PSNU/SNA, especially considering that we live in an increasingly digital world. We can analyze the ways and contexts (e.g., not while doing occupational or social activities, not at night, especially just before going to sleep) in which social networks could be used in a healthier way and in line with personal values. Therefore, interventions, such as digital literacy programs and mental health interventions for adolescents and young adults could be implemented at various levels of prevention, considering their impact on development [6].

## 5. Conclusions

Our results suggest that FoMO and experiential avoidance are relevant variables for understanding PSNU and gender differences in young adults. FoMO correlates with PSNU in the overall sample, and in both men and women, and contributes more (than the other variables) to the variance in PSNU, both in the total sample and in women. In addition, the PSNU group scores higher on FoMO than the WPSNU group. The fact that FoMO is an important variable in explaining PSNU is not new. The contribution of our work is that, when compared to variables such as anxiety, depression, or stress, FoMO has greater predictive power in PSNU, mainly in women.

In terms of experiential avoidance, we found significant differences according to gender, with higher scores in women, and it was positively related to PSNU in the total sample, in women, and in men. In addition, the PSNU group had significantly higher scores than the WPSNU group in experiential avoidance. These results support the idea that PSNU may be a strategy for avoiding distress, specifically FoMO. This fear seems to be fueled by the feeling that certain psychological needs (e.g., need for self-presentation, assertiveness, belonging, autonomy, competence, and relationships) are met in the virtual world but not in the real world [17,28,40]. Given the gender differences found, it would be interesting to investigate the dynamics and reasons why men and women use social media and why they end up using them problematically [24].

## Figures and Tables

**Table 1:** Sociodemographic characteristics.

Variables	Total (*N* = 219)	Men (*n* = 56)	Women (*n* = 163)	*t* or χ^2^	*p*	Cohen’s *d*	CI for *d*	
							LB	UB
Age *M* (*SD*)	20.5 (2.42)	21.15 (2.27)	20.28 (2.19)	2.501	**0.013** *	0.329	0.082	0.700
Education *n* (*%*)				3.748	0.441			
Elementary School	1 (0.5)	0	1 (0.6)					
Middle School	1 (0.5)	1 (1.8)	0					
High School	85 (38.8)	23 (41.8)	62 (38)					
Undergraduate	126 (57.5)	30 (53.6)	96 (58.9)					
Graduate	6 (2.7)	2 (3.6)	4 (2.5)					
Hours per day of SNU *M (SD)*								
Monday to Friday	4.08 (1.78)	4.10 (1.93)	4.06 (1.74)	0.105	0.917	0.017	−0.306	0.341
During weekend	4.91 (2.64)	5.06 (2.87)	4.87 (2.87)	0.115	0.653	0.074	−0.247	0.394

SNU: Social networks use; * *p* < 0.05.

**Table 2:** Means (standard deviations) and comparison of means for fear of missing out, experiential avoidance, anxiety, depression, and stress. Student’s *t*-test for gender.

Variables (Instruments)	Total (*N* = 219)			Men (*n* = 56)			Women (*n* = 163)			*t*	*p*	Cohen’s *d*	95% for CI	
	*M* (*SD*)	Min	Max	*M* (*SD*)	Min	Max	*M* (*SD*)	Min	Max				LB	UB
PSNU (TARS)	11.80 (5.15)	1	28	11.42 (5.37)	1	26	11.93 (5.08)	2	28	−0.640	0.523	−0.099	−0.403	0.205
Fear of missing out (FoMO)	22.17 (7.94)	10	50	22.09 (7.25)	11	41	22.20 (8.19)	10	50	−0.091	0.927	−0.014	−0.318	0.289
Experiential avoid. (AAQ-II)	24.86 (10.03)	7	49	21.74 (9.01)	7	41	25.94 (10.16)	7	49	−2.74	**0.007**	**−0.425**	−0.730	−0.118
Anxiety (DASS-21)	4.15 (3.79)	0	16	3.33 (3.30)	0	14	4.44 (3.92)	0	16	−1.903	0.058	−0.295	−0.599	0.010
Depression (DASS-21)	4.44 (4.60)	0	21	3.97 (4.07)	0	16	4.60 (4.77)	0	21	−0.878	0.381	−0.136	−0.440	0.168
Stress (DASS-21)	6.58 (4.60)	0	20	4.91 (4.09)	0	15	7.15 (4.66)	0	20	−3.191	**0.002**	**−0.494**	−0.801	−0.187

PSNU: Problematic social network use; TARS: Social Networks Addiction Test (“Test de Adicción a las Redes Sociales”); avoid.: avoidance; AAQ-II: Acceptance and Action Questionnaire-II; DASS-21: Depression, Anxiety and Stress Scales. Significant *p*-values are in bold. Significant values are in bold. Size effect (Cohen’s *d*): <0.20 trivial; >0.20 and <0.50, small; >0.50 and <0.80, medium; >0.80, large.

**Table 3:** Pearson’s correlation for the total sample.

Variables (Instruments)	1	2	3	4	5	6
1. PSNU (TARS)	--	0.428 ***	0.217 ***	0.139 *	0.173 *	0.155
2. Fear of missing out (FoMO)		--	0.383 ***	0.269 ***	0.308 ***	0.317 ***
3. Experiential avoidance (AAQ-II)			--	0.507 ***	0.624 ***	0.578 ***
4. Anxiety (DASS-21)				--	0.721 ***	0.676 ***
5. Depression (DASS-21)					--	0.699 ***
6. Stress (DASS-21)						--

*N* = 219. * *p* ≤ 0.05; *** *p* ≤ 0.001.

**Table 4:** Pearson’s correlation for women.

Variables (Instruments)	1	2	3	4	5	6
1. PSNU (TARS)	--	0.441 ***	0.164 *	0.078	0.126	0.131
2. Fear of missing out (FoMO)		--	0.399 ***	0.250 ***	0.274 ***	0.319 ***
3. Experiential avoidance (AAQ-II)			--	0.492 ***	0.640 ***	0.568 ***
4. Anxiety (DASS-21)				--	0.728 ***	0.654 ***
5. Depression (DASS-21)					--	0.683 ***
6. Stress (DASS-21)						--

*n* = 163. * *p* ≤ 0.05; *** *p* ≤ 0.001.

**Table 5:** Pearson’s correlation for men.

Variables (Instruments)	1	2	3	4	5	6
1. PSNU (TARS)	--	0.392 **	0.369 **	0.324 *	0.319 *	0.208
2. Fear of missing out (FoMO)		--	0.351 **	0.354 **	0.441 ***	0.338 *
3. Experiential avoidance (AAQ-II)			--	0.512 ***	0.568 ***	0.536 ***
4. Anxiety (DASS-21)				--	0.689 ***	0.728 ***
5. Depression (DASS-21)					--	0.783 ***
6. Stress (DASS-21)						--

*n* = 56. * *p* ≤ 0.05; ** *p* ≤ 0.01; *** *p* ≤ 0.001.

**Table 6:** Multiple regression analysis for problematic social network use (TARS) with total sample.

Model DV: PSNU	Unstandar. Coeff.		Standar. Coeff.	*t*	*p*	95% CI for B		VIF
	B	SE	β			LB	UB	
FoMO	0.262	0.044	0.404	6.016	**<0.001**	0.176	0.348	1.186
Experiential avoidance	0.027	0.042	0.053	0.649	0.517	−0.056	0.110	1.769
Anxiety	−0.023	0.122	−0.017	−0.191	0.849	−0.263	0.216	2.110
Depression	0.031	0.111	0.028	0.282	0.778	−0.187	0.249	2.569

*n* = 219. DV: Dependent variable; Unstandar.: Unstandardized; Coeff.: coefficients; Standar.: Standardized; SE: Standard error; CI: Confidence interval; LB: Lower bound; UB: Upper bound; VIF: Variance inflation factor. Significant values are in bold.

**Table 7:** Multiple regression analysis for problematic social network use (TARS) with women sample.

Model DV: PSNU	Unstandar. Coeff.		Standar. Coeff.	*t*	*p*	95% CI for B		VIF
	B	SE	β			LB	UB	
FoMO	0.278	0.048	0.447	5.785	**<0.001**	0.183	0.372	1.189
Experiential avoidance	−0.007	0.039	−0.015	−0.193	0.847	−0.084	0.069	1.189

*n* = 163. DV: Dependent variable; PSNU: Problematic social network use; Coeff.: coefficients; SE: Standard error; CI: Confidence interval; LB: Lower bound; UB: Upper bound; VIF: variance inflation factor. Significant *p*-values are in bold.

**Table 8:** Multiple regression analysis for problematic social network use (TARS) with men sample.

Model DV: PSNU	Unstandar. Coeff.		Standar. Coeff.	*t*	*p*	95% CI for B		VIF
	B	SE	β			LB	UB	
FoMO	0.206	0.103	0.277	1.998	0.051	−0.001	0.412	1.268
Experiential avoidance	0.128	0.092	0.214	1.387	0.171	−0.057	0.312	1.563
Anxiety	0.200	0.283	0.123	0.707	0.483	−0.368	0.768	1.989
Depression	−0.013	0.246	−0.010	−0.051	0.960	−0.507	0.482	2.289

*n* = 56.

**Table 9:** Means (standard deviations) and comparison of means for groups with and without problematic social network use.

Variables (Instruments)	PSNU Group (*n* = 60)	WPSNU Group (*n* = 159)	*t*	*p*	Cohen’s *d*	95% CI for *d*	
						LB	UB
Hours per day of SNU (Monday to Friday)	4.75 (1.81)	3.81 (1.71)	3.463	**<0.001**	0.540	0.229	0.850
Hours per day of SNU(during weekend)	6.11 (2.61)	4.43 (2.51)	4.278	**<0.001**	0.659	0.350	0.967
Fear of missing out (FoMO)	26.12 (7.80)	20.68 (7.50)	4.726	**<0.001**	0.716	0.411	0.020
Experiential avoidance (AAQ-II)	27.61 (9.31)	23.83 (10.12)	2.521	**0.012**	0.382	0.082	0.681
Anxiety (DASS-21)	4.47 (3.68)	4.04 (3.84)	0.752	0.453	0.144	−0.183	0.411
Depression (DASS-21)	5.22 (5.06)	4.14 (4.41)	1.555	0.121	0.236	−0.063	0.533
Stress (DASS-21)	6.88 (4.28)	6.46(4.75)	0.596	0.552	0.090	−0.207	0.387

Significant *p*-values are in bold. Size effect (Cohen’s *d*): <0.20 trivial; >0.20 and <0.50, small; >0.50 and <0.80, medium; >0.80, large.

## Data Availability

The data presented in this study are available on request from the corresponding author due to the nature of the questionnaires, which assessed personal and sensitive information.

## Supplementary Materials

The following supporting information can be downloaded at: https://www.mdpi.com/article/10.3390/healthcare13222988/s1, Figure S1: Scatter Plot for total sample; Figure S2: Normal Q-Q Plot for total sample; Figure S3: Scatter Plot for women sample; Figure S4: Normal Q-Q Plot for women sample; Figure S5: Scatter Plot for men sample; Figure S6: Normal Q-Q Plot for men sample.

## Abbreviations

The following abbreviations are used in this manuscript:

FoMO	Fear of missing out
PSNU	Problematic social network use
SNA	Social network addiction
WPSNU	Without problematic social network use
