# Supplementary material for: Are FoMO, Experiential Avoidance, and Emotional Distress Related to Problematic Social Network Use in Young Adults?
Source: Healthcare (Basel). 2025 Nov 20;13(22):2988. doi: 10.3390/healthcare13222988 (PMC12652083; doi:10.3390/healthcare13222988)

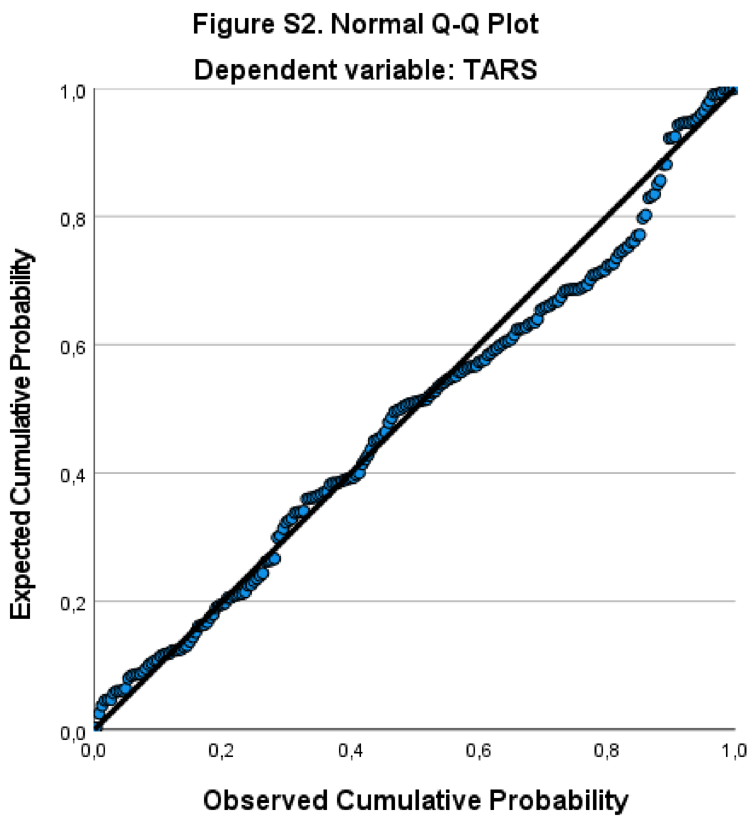
Figure S2. Normal Q-Q Plot
Dependent variable: TARS
Expected Cumulative Probability
1,0
0,8
0,6
0,4
0,2
0,0
0,0
0,2
0,4
0,6
0,8
1,0
Observed Cumulative Probability

Figure S2. Normal Q-Q Plot
Dependent variable: TARS

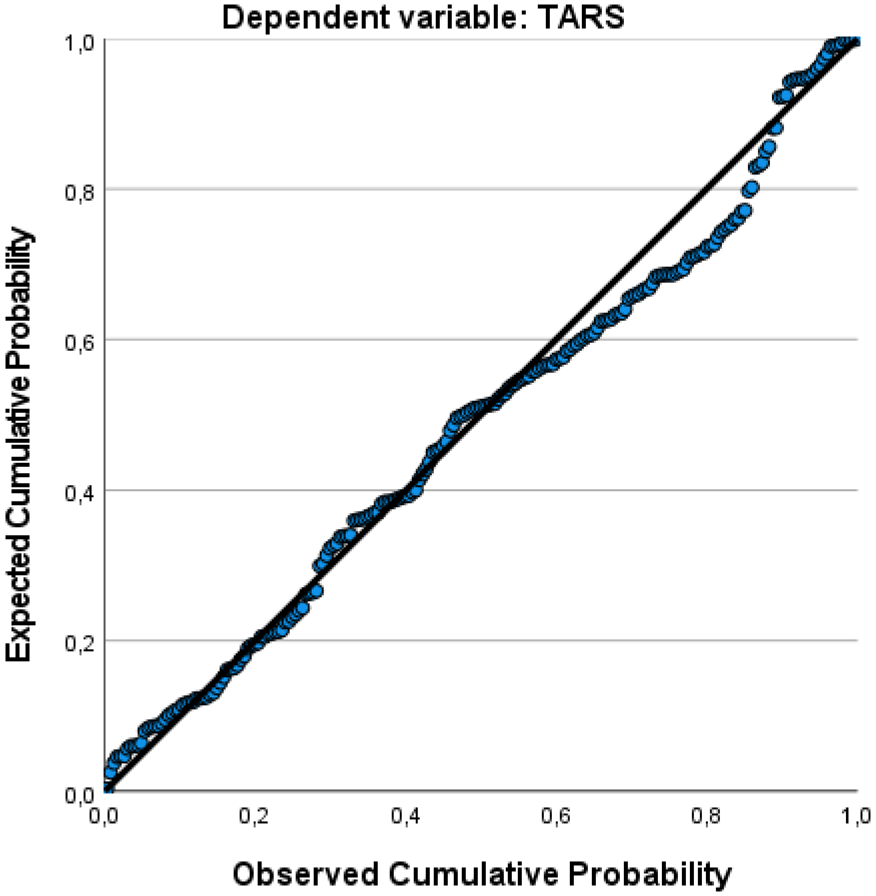

Supplement: Supplementary file 1 [file healthcare-13-02988-s001.zip › Suplementary materials/Figure S2. Normal Q-Q Plot for total sample.pdf]
