# Supplementary figures and images for: Are FoMO, Experiential Avoidance, and Emotional Distress Related to Problematic Social Network Use in Young Adults?
Source: Healthcare (Basel). 2025 Nov 20;13(22):2988. doi: 10.3390/healthcare13222988 (PMC12652083; doi:10.3390/healthcare13222988)

Figure S1. Scatter Plot  
Dependent variable: TARS

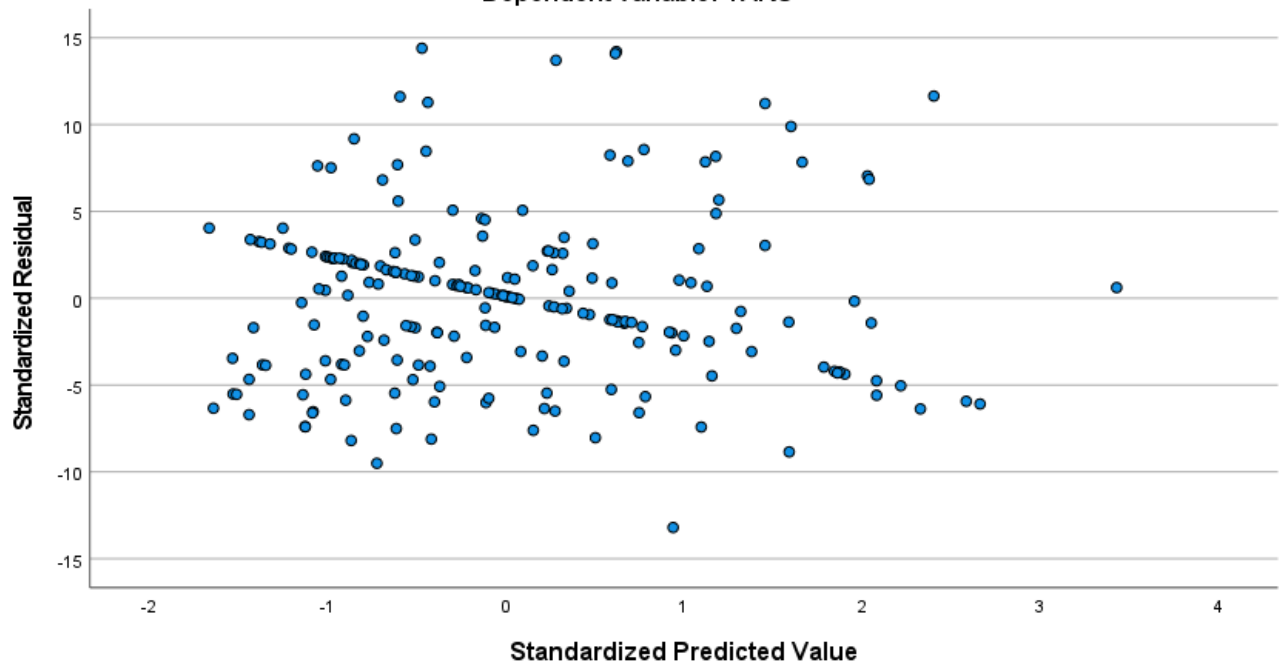

Supplement: Supplementary file 1 [file healthcare-13-02988-s001.zip › Suplementary materials/Figure S1. Scatter Plot for total sample.pdf]

Figure S3. Scatter Plot  
Dependent variable: TARS

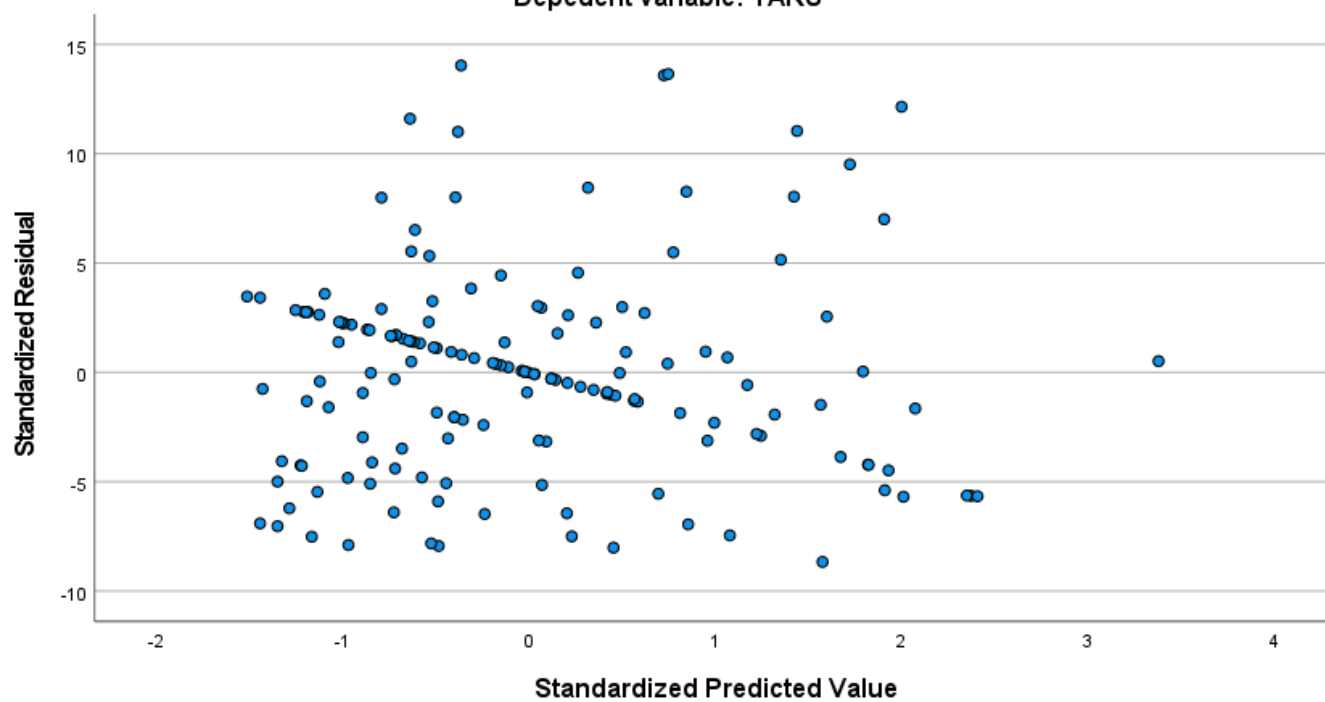

Supplement: Supplementary file 1 [file healthcare-13-02988-s001.zip › Suplementary materials/Figure S3. Scatter Plot.pdf]

Figure S4. Normal Q-Q Plot  
Dependent variable: TARS

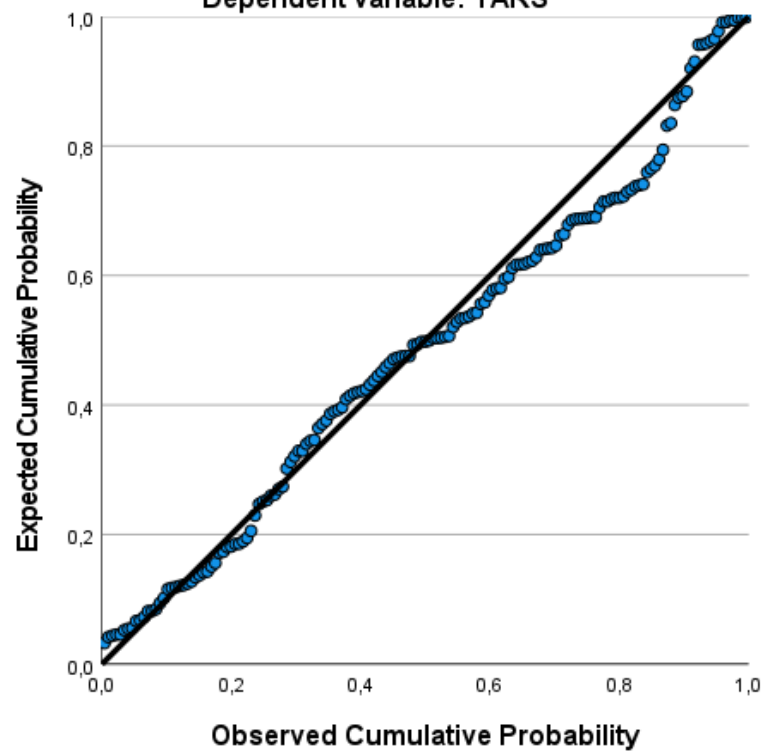

Supplement: Supplementary file 1 [file healthcare-13-02988-s001.zip › Suplementary materials/Figure S4. Normal Q-Q Plot.pdf]

Figure S5. Scatter Plot  
Dependent variable: TARS

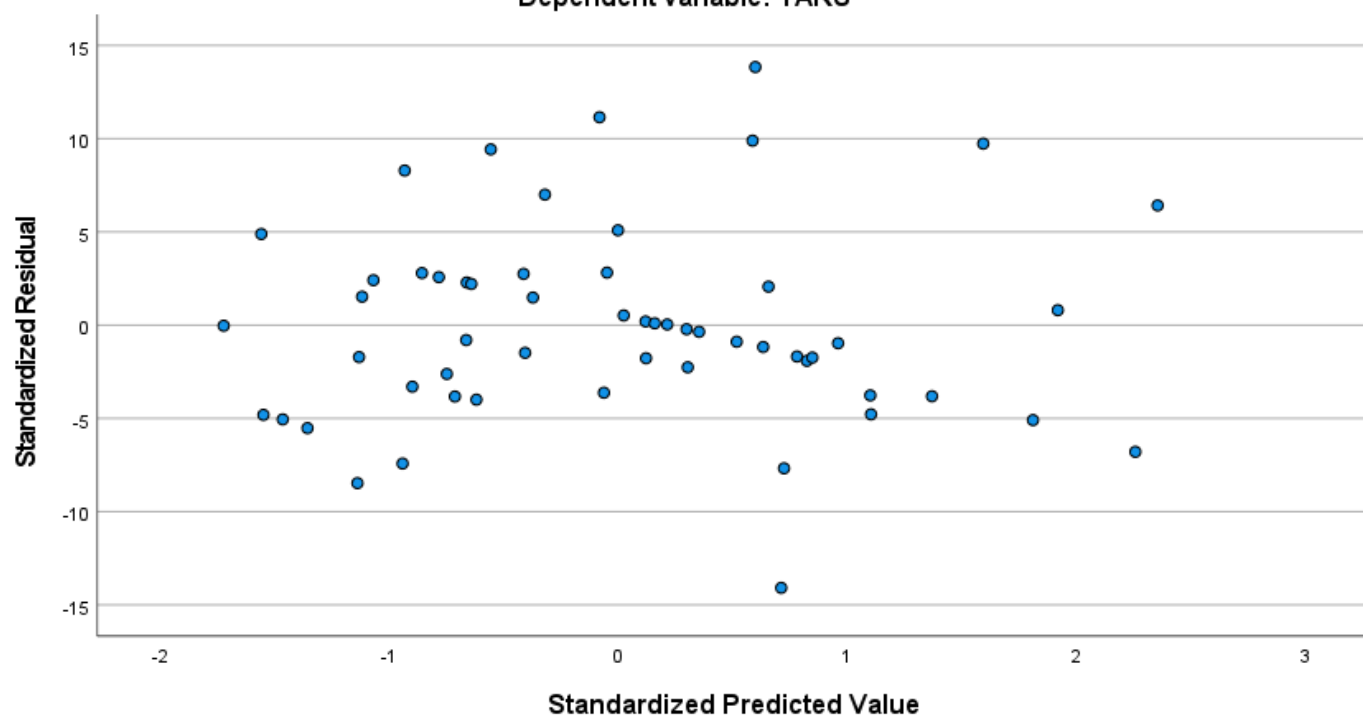

Supplement: Supplementary file 1 [file healthcare-13-02988-s001.zip › Suplementary materials/Figure S5. Scatter Plot.pdf]

Figure S6. Normal Q-Q Plot  
Dependent variable: TARS

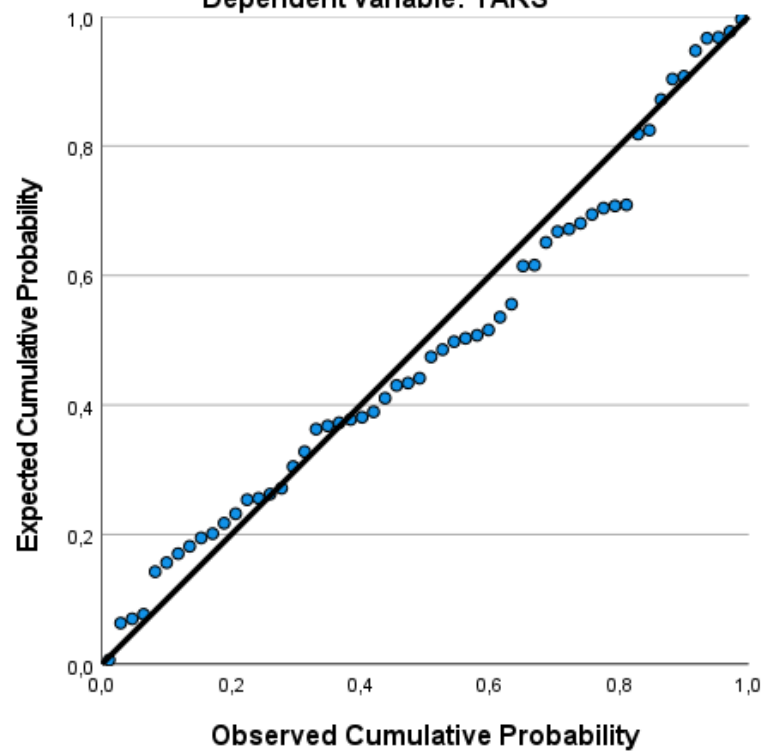

Supplement: Supplementary file 1 [file healthcare-13-02988-s001.zip › Suplementary materials/Figure S6. Normal Q-Q Plot.pdf]
